# Mutational Evolution in Relapsed Diffuse Large B-Cell Lymphoma

**DOI:** 10.3390/cancers10110459

**Published:** 2018-11-20

**Authors:** Marcel Nijland, Annika Seitz, Martijn Terpstra, Gustaaf W. van Imhoff, Philip M Kluin, Tom van Meerten, Çiğdem Atayar, Léon C. van Kempen, Arjan Diepstra, Klaas Kok, Anke van den Berg

**Affiliations:** 1 Department of Hematology, University of Groningen, University Medical Center Groningen, 9713 GZ Groningen, The Netherlands; g.w.van.imhoff@umcg.nl (G.W.v.I.); t.van.meerten@umcg.nl (T.v.M.); 2 Department of Pathology and Medical Biology, University of Groningen, University Medical Center Groningen, 9713 GZ Groningen, The Netherlands; a.seitz@umcg.nl (A.S.); p.m.kluin@umcg.nl (P.M.K.); l.van.kempen@umcg.nl (L.C.v.K.); a.diepstra@umcg.nl (A.D.); a.van.den.berg01@umcg.nl (A.v.d.B.); 3 Department of Genetics, University of Groningen, University Medical Center Groningen, 9713 GZ Groningen, The Netherlands; m.m.terpstra.cluster@gmail.com (M.T.); k.kok@umcg.nl (K.K.); 4 Department of Pathology, Treant Caregroup, Bethesda Hospital, 7909 AA Hoogenveen, The Netherlands; c.atayar@treant.nl

**Keywords:** diffuse large B-cell lymphoma, relapse, mutations, heterogeneity, evolution, fresh frozen paraffin embedded

## Abstract

Current genomic models in diffuse large B-cell lymphoma (DLBCL) are based on single tumor biopsies, which might underestimate heterogeneity. Data on mutational evolution largely remains unknown. An exploratory study using whole exome sequencing on paired (primary and relapse) formalin fixed paraffin embedded DLBCL biopsies (*n* = 14) of 6 patients was performed to globally assess the mutational evolution and to identify gene mutations specific for relapse samples from patients treated with rituximab, cyclophosphamide, doxorubicin, vincristine, and prednisolone. A minority of the mutations detected in the primary sample (median 7.6%, range 4.8–66.2%) could not be detected in the matching relapse sample. Relapsed DLBCL samples showed a mild increase of mutations (median 12.5%, range 9.4–87.6%) as compared to primary tumor biopsies. We identified 264 genes possibly related to therapy resistance, including tyrosine kinases (*n* = 18), (transmembrane) glycoproteins (*n* = 73), and genes involved in the JAK-STAT pathway (*n* = 7). Among the potentially resistance related genes were *PIM1*, *SOCS1*, and *MYC*, which have been reported to convey a risk for treatment failure. In conclusion, we show modest temporal heterogeneity between paired tumor samples with the acquisition of new mutations and identification of genes possibly related to therapy resistance. The mutational evolution could have implications for treatment decisions and development of novel targeted drugs.

## 1. Introduction

Diffuse large B-cell lymphoma (DLBCL) accounts for 25–35% of all non-Hodgkin lymphomas (NHL) [1]. For more than 15 years, immuno-chemotherapy with rituximab, cyclophosphamide, doxorubicin, vincristine, and prednisolone (R-CHOP) has been the standard of care [2]. Although the prognosis for patients with low-risk disease is excellent, the 3-years overall survival (OS) for high-risk patients is less than 65% [3]. Patients with primary refractory disease or those who relapse within a year after treatment have an especially poor response to salvage chemotherapy [4,5].

DLBCL is a genetically heterogeneous disease. Based on gene expression profiling (GEP), DLBCL can be subdivided into activated B-cell (ABC-type), germinal center B-cell (GCB-type), and unclassified-type. The first two subtypes reflect the B-cell developmental stages from which DLBCL arises [6]. The prognostic impact of this so-called cell-of-origin (COO) classification based on gene expression profiles (GEP) has been established in multiple studies [6,7]. Several trials are currently investigating the efficacy of adding compounds targeting the putative mutations or deregulated molecular pathways associated with a specific COO class; for example, adding ibrutinib or lenalidomide to R-CHOP therapy in patients with ABC subtype DLBCL [6,7,8].

Over the last decade, a large number of studies have been published on the mutational landscape of DLBCL, including over 2000 DLBCL cases [9,10,11,12,13,14,15,16,17,18,19,20,21]. These studies identified non-synonymous mutations in 30 to 100 genes per case (median 3.3 to 6.6 mutations per megabase) [21]. In total, over 1000 individual mutated genes have been described. The mutational landscape differs between ABC-type and GCB-type, with mutations in *MYD88* and *CD79B* being more common in the ABC-type, and mutations in *EZH2* and *GNA13* being more common in GCB-type [9,10,11,12,13,14,15,16,17,18,19,20,21]. Based on the mutational landscapes, DLBCL can be divided into subgroups characterized by genetic alterations in the proximal B-cell receptor, NF-kB signaling, PI3-kinase signaling, anti-apoptotic proteins, DNA damage repair, and immune evasion [20,21].

One of the main challenges for most genomic profiles is to implement them into clinical practice. Due to the high inter-patient heterogeneity of mutations, it has been estimated that for the development of a prognostic model, the mutational landscape of 900 patients would have to be correlated with clinical outcomes [22]. Earlier trials had been underpowered to properly address this question [16,18]. More recently, larger studies have reported on the prognostic impact of genomic risk models [19,20,21]. Apart from being prognostic, these genomic models can form the basis for biomarker driven treatment strategies [23].

Most of the currently published papers do not take into account genomic evolution of tumors with presence of subclones at different anatomical sites (spatial), evolution over time (temporal), and dynamics caused by treatment [24]. At this point, data on the clonal evolution of DLBCL is largely absent. Data on sequential biopsies in DLBCL is available for less than 20 patients [15,18].

Thus, despite the large number of DLBCL samples analyzed, the impact of clonal and mutational evolution remains largely unknown. To broaden this knowledge we performed an exploratory study on paired biopsies (primary versus relapse) to globally assess the mutational evolution, and to identify gene mutations enriched or exclusively present in relapsing patients.

## 2. Materials and Methods

### 2.1. Patient Selection 

All patients diagnosed with a relapsed or refractory (R/R) DLBCL between 2004 and 2014 at the University Medical Center Groningen (UMCG) or affiliated hospitals were retrieved from the electronic database of the UMCG. Only patients that received 6 to 8 cycles of R-CHOP as first line therapy were included. Patients with post-transplant lymphoproliferative disease, human immunodeficiency virus (HIV) related lymphoma, primary central nervous system lymphoma (CNS), primary testicular lymphoma, primary mediastinal large B-cell lymphoma, or transformed indolent lymphoma were excluded. Of the 61 patients with R/R DLBCL, a histological confirmation of relapse was available for 31 patients. CNS relapse was confirmed by flow cytometry on spinal fluid samples in 7 patients. In the remaining 23 patients, progression was established through imaging modalities. Of the 31 patients with a histological proven relapse, 16 had an excision biopsy sufficient for further analyses (Figure S1). The remainder of the patients had core or bone marrow biopsies insufficient for analysis. Where applicable, archival samples of non-tumor tissue were retrieved for isolation of germ line DNA. Approval for this non interventional study was obtained from the Medical Ethics Review Committee from the University Medical Center Groningen (October 2014). Informed consent was waived in accordance with Dutch regulations The study utilized rest material from patients, the use of which is regulated under the code for good clinical practice in the Netherlands and does not require informed consent in accordance with Dutch regulations.

### 2.2. Pathology Review 

Pathology review was performed according to the 2017 “WHO classification of tumors of haematopoietic and lymphoid tissues” on formalin fixed paraffin embedded (FFPE) biopsies by an experienced hemato-pathologist (AD) [1]. For COO classification, raw counts obtained by nanostring gene expression analysis were uploaded at the Lymphoma/Leukemia Molecular Profiling Project (LLMPP) website for COO categorization (https://llmpp.nih.gov/LSO/LYMPHCX/lymphcx_predict.cgi) [7].

### 2.3. Fluorescence in Situ Hybridization

*MYC* rearrangements were assessed on interphase nuclei on 3 µm thick whole tissue sections of the primary tumor as previously described, with Vysis break apart probes (Abbot Technologies, Santa Clara, CA, USA) using fluorescence in situ hybridization (FISH) [25]. All cases with a *MYC* break were also analyzed for BCL2 and BCL6 breaks using Vysis break apart probe assays (Abbot Technologies, Santa Clara, CA, USA). 

### 2.4. DNA Isolation

In total, 67 FFPE tissue blocks were obtained from 16 DLBCL patients. For whole exome sequencing (WES) we selected tumor samples with at least 50% tumor cells. To yield at least 500 µg DNA, a minimum area of 0.5 cm^2^ of tumor cells was obtained from 10 µm thick slides. DNA from FFPE tumor and non-tumor biopsies was isolated using the QIAamp DNA FFPE tissue kit (Qiagen, Hilden, Germany), following the protocol of the manufacturer. A standard salt-chloroform protocol was used to isolate DNA from stem cells collected for hematopoietic stem cell transplantation (CD34+ purified cells) from one patient. DNA concentrations were measured by NanoDrop (Thermo Fisher Scientific Inc., Waltham, MA, USA), and DNA quality was evaluated on a 1% agarose gel. After quality control, 59 samples from 15 patients were sent for WES (Figure S1).

### 2.5. Whole Exome Sequencing 

Library preparation and whole exome sequencing was carried out by Novogene (Novogene Bioinformatics Technology Co., Ltd, Beijing, China). Library preparation was done using the Agilent SureSelect All Exon V6 kit (Agilent Technologies, Santa Clara, CA, USA), starting from 0.5–1.5 μg genomic DNA of tumor and non-tumor samples. Paired-end sequencing with a read length of 2 × 100 nucleotide was performed on Illumina HiSeq2000 (Illumina, Inc., San Diego, CA, USA).

### 2.6. Bioinformatics Approach

The bioinformatics pipeline of the UMCG genome facility was used for data analysis, as described previously [26,27]. Briefly, reads were aligned to the human 1000 genomes reference based on the GRCh37 build using BWA 5.9rc. [28]. Picard tools were used for format conversion and marking duplicate reads. The Genome Analysis Toolkit (GATK1) was used for realignment of insertions and deletions (Indels), and Molgenis Compute 4 for base score quality recalibration (BSQR) [29,30]. Custom scripts in the VCF tools library were used to generate VCF files, variant calling was performed using the GATK unified genotype, and variant annotation using snpeff/snpsift 3.5 with the ensemble release 74 gene annotations (http://www.ensembl.org/index.html), dbNSFP2.3, and GATK with annotations from the Database of Single Nucleotide Polymorphisms (dbSNP) Bethesda (MD), National Center for Biotechnology Information, National Library of Medicine (dbSNP Build ID: 137), and CosmicCodingMuts_v62. [31,32,33,34].

To identify reliable somatic mutations, variants with total reads <20× in either the normal, primary, or resistant samples were excluded. In addition, we excluded all variants with ≥2 mutant reads in the normal sample, as these might represent personal variants. The remaining variants were aligned against the Exome Aggregation Consortium (ExAC) database (Broad Institute, Cambridge, MA; URL: http://exac.broadinstitute.org) to screen for any remaining known single nucleotide polymorphisms. In addition, we removed variants that (1) were present in the Caucasian based 1000-Genome with an allele frequency larger than 0.2%, (2) map in noncoding regions, (3) were synonymous, (4) have a quality score <20, or (5) have a mapping quality <20. Only variants with ≥2 mutant reads were taken into account. 

### 2.7. Genes Possibly Related to Therapy Resistance

Variants specific for the R/R, and variants with mutant read frequencies (MAFs) in the resistant samples ≥20% and with a MAF at least two times higher compared to the MAF in the paired primary sample, were indicated as “*possibly related to therapy resistance*”, provided the tumor cell percentage in primary and relapse samples was similar. 

## 3. Results

### 3.1. Patient Characteristics 

Of the 59 samples originating from 15 patients sent for WES, library preparation failed in all 6 samples of a single patient. The average sequencing coverage of the remaining patients was insufficient (<20×) in either the primary sample (6 patients), the relapse sample (1 patient), or both samples (1 patient) (Figure S1). In total, WES data of sufficient quality from sequential biopsies was obtained for six patients. For two of the six patients with relapse biopsies from different anatomical sites, both biopsies were taken at the same time for one patient and at different time points for the other patient. Patient and clinicopathological characteristics are summarized in Table 1. A *MYC* and a *BCL6* rearrangement were observed in the primary tumor sample of patient 5. In 5 patients, relapses occurred within 24 months from diagnosis, and one patient had a relapse at 55 months. The five patients that died were all due to lymphoma progression.

### 3.2. Quality Control

The median read depth of the WES data of the 14 tumor samples was 85× (range 29–203×) (Figure S2). The median number of non-synonymous coding single nucleotide variants (SNVs) and Indels per genome was 568 (range 77–949), affecting 1896 genes in total. An estimation of the admixture of normal cells based on the mean mutant allele frequency (MAF) of all somatic mutations in the 25–75% interquartile range, as previously described [26], revealed tumor cell percentages ranging from 78 to 92% (median 90%), and is in concordance with the pathologist’s (AD) estimation (data not shown). No significant differences were observed between tumor cell percentages in the primary and relapse samples (Figure S3). The mean read depth of genes frequently mutated in DLBCL was 285× (range 59–1010×) (Table S1). For a few exons, the depth was insufficient to reliably assess the presence of mutations. In particular, this was the case for exon 3 of FOXO1, with no reads in any of the samples.

### 3.3. Commonly Mutated Genes

**Figure 1:**
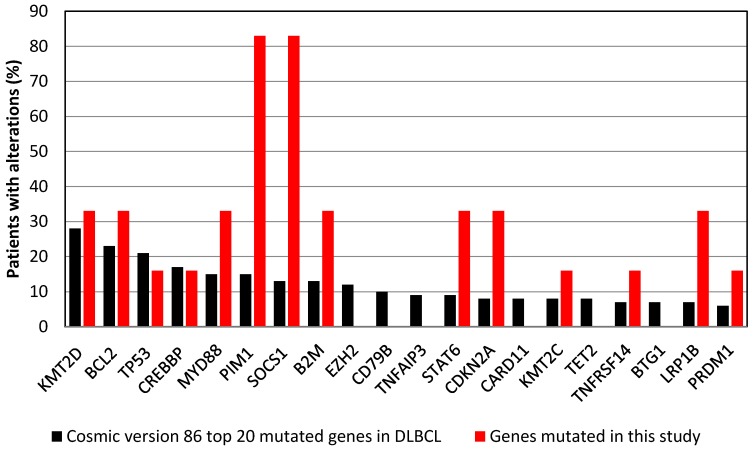
Frequency of mutations in the top-20 most commonly mutated genes in diffuse large B-cell lymphoma according to the Cosmic database version 86, and as observed in the 14 tumor samples analyzed in this study. Fourteen of the 20 genes were mutated in at least one of the 14 samples. *SOC1* and *PIM1* mutations were observed in 5 of 6 patients.

Fourteen of the 20 genes most frequently mutated in DLBCL according to the Cosmic database (version 86) were mutated in one or more of the cases in our study (Figure 1). We identified 28 genes with mutations in at least 3 patients (Table S2). Functional annotation of these genes showed enrichment for genes involved in antigen presentation, including the human leukocyte antigen (HLA) molecules and immunoglobulin light chains. Mutations in Suppressor of Cytokine Signaling 1 (*SOCS1*) and Pim-1 Proto-Oncogene (*PIM1*) were observed in 5 out of 6 patients (Figure S4). The patient with a *MYC* rearrangement had 3 missense mutations in exon 2 of the *MYC* gene. Furthermore, four additional *MYC* mutations were found in two patients without a *MYC* rearrangement (Figure S4). 

### 3.4. Mutational Evolution

**Figure 2:**
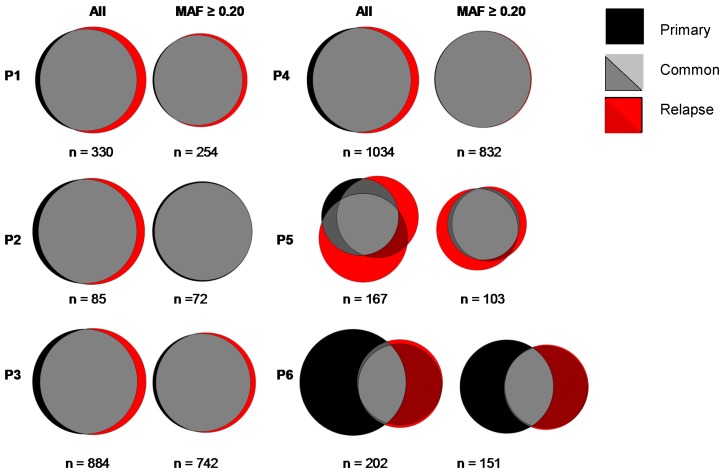
Venn diagrams showing for each individual patient (P1–6) the overlap in mutations between primary and paired relapse tumor samples. Left panels are Venn diagrams for all mutations (all), and right panels are Venn diagrams for mutations with a mutant allele frequency (MAF) ≥0.2. The total numbers of mutations per patient are depicted below each diagram. The size of the relapse diagram is proportional to the primary sample. In the patients with two biopsies at relapse (P5 and P6), the concordance between the novel mutations in the relapse samples was 45.3% and 89.2%, indicative of spatial heterogeneity.

With the exception of one patient, the vast majority of the mutations were shared between the primary and R/R samples. The median percentage of mutations detected in the primary and not in the matching relapse sample was 7.6% (range 4.8–66.2%) (Figure 2, Table S3). The loss of mutations was particularly high in the patient with a late relapse, where 66% of mutations detected in the primary sample could not be detected in the relapse sample. The mean MAF of the mutations only detected in primary samples was 0.15, which is in the lower quartile of the distribution, indicating genomic heterogeneity in the tumor cells.

Relapsed DLBCL samples showed a median increase of non-synonymous mutations of 12.5% (range 9.4–87.6%) as compared to primary tumor biopsies (Figure 2, Table S3). In the two patients with multiple biopsies at relapse, there was a 45.3% and 89.2% concordance for mutations detected only in the relapse samples (Figure 2). There was no significant correlation between the loss or increase in mutations and time until relapse (ρ 0.32; p, 0.71) (Figure S5). Mutations were randomly distributed across the genome (Figure S6). 

Of the 354 relapse specific mutations (in 303 genes), 195 (55%) had a MAF <0.2 and were probably subclonal. The remaining 159 (45%) mutations had MAF ≥0.2 and are probably major clone mutations, and thus possibly related to therapy resistance (Supplementary Table S3). In addition, we identified 215 mutations that showed at least a two-fold increase in MAF in the relapse biopsy compared to the primary sample, indicating a possible relation with therapy. The combined set of 374 mutations possibly related to therapy resistance encompassed 264 genes (Figure S7). Functional annotation of these genes revealed 18 tyrosine kinases, 73 (transmembrane) glycoproteins, and 7 genes that are related to the JAK-STAT pathway (Table S4). 

Several of the genes with relapse specific mutations are known to be targets for somatic hypermutation, including *BCL2*, *BIRC3*, *BTG2*, *IRF4*, *MYC*, *PIM1*, *SGK1*, and *SOCS1* [21,35]. The most frequently observed base substitution among the relapse specific mutations (C:G > T:A) is a known cyclophosphamide-induced base substitution, and to a lesser extent a canonical Activation-Induced Deaminase (AID) activity dependent substitution (Figure S8) [35,36].

**Figure 3:**
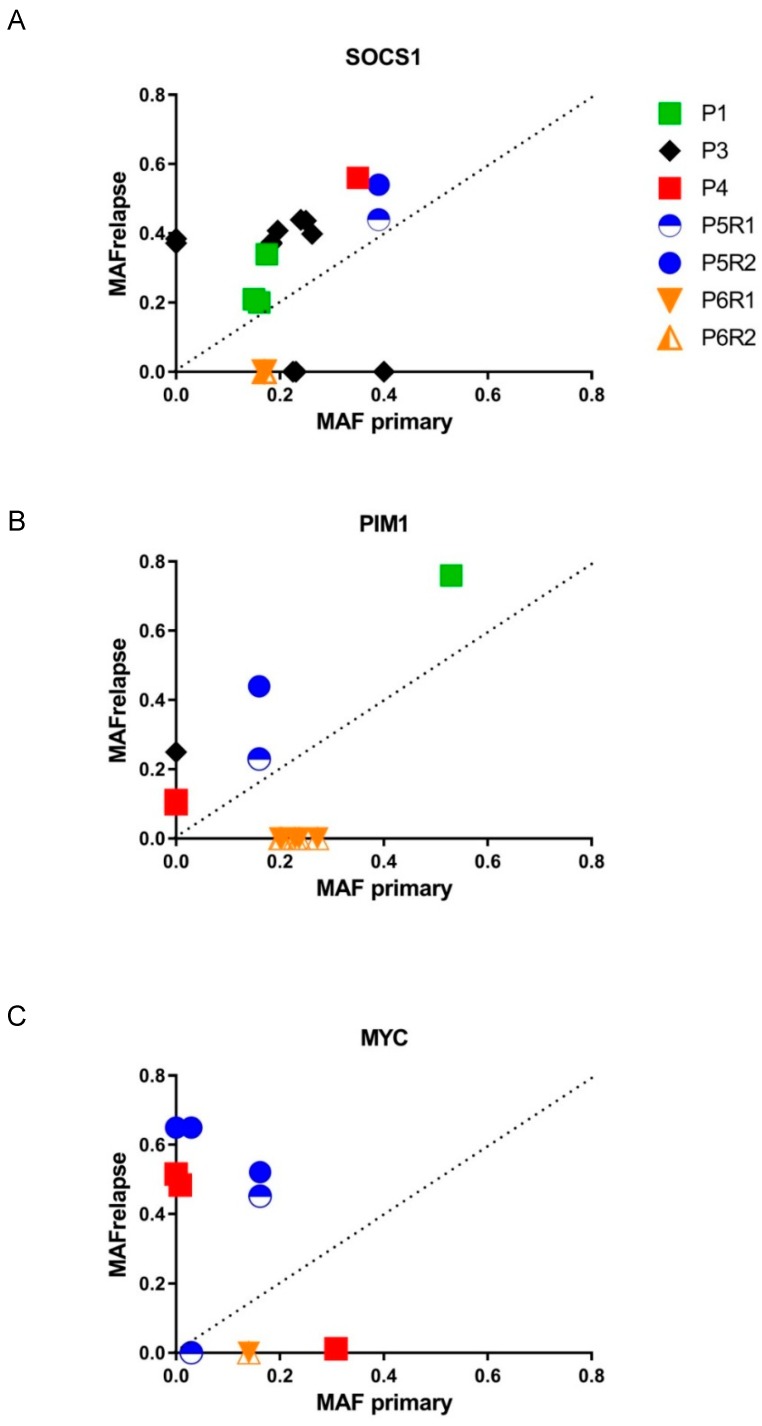
Graphical representation of mutant allele frequency (MAF) of (**A**) *SOCS1*, (**B**) *PIM1*, and (**C**) *MYC* in paired biopsies. Mutations above the dashed line are considered as possibly related to therapy resistance. The MAF of *SOCS1* mutations remains relatively stable. The MAF of mutations in *PIM1* and *MYC* had at least a two-fold increase in 2 of 5 and 2 of 3 patients, respectively.

The evolution of the resistance-associated and other mutations in *SOCS1, PIM1*, and *MYC* showed different patterns (Figure 3). The MAF of mutations in *SOCS1* showed moderate increases in relapse samples. Mutations in *PIM1* showed at least two-fold increased MAF in two out of five patients. In the patients with two relapse biopsies, the MAF of *SOCS1* and *PIM1* were similar across the relapse samples (Figure 3A,B). Even in this small cohort, the dynamics of *MYC* mutations showed clear heterogeneity with loss of mutations (*n* = 3), increase of MAF (*n* = 1), and gain of mutations (*n* = 3) in relapse samples (Figure 3C).

## 4. Discussion

Mutational analysis has expanded the knowledge on the pathogenesis of DLBCL with genomic risk models that can aid future biomarker driven treatment strategies [19,20,21]. However, mutational analysis from single tumor biopsies will underestimate the true genomic landscape of tumors due to the presence of inter- and intra-tumor heterogeneity, natural clonal evolution, and therapy related changes [24,37]. Through targeted sequencing of the variable, diversity and joining (VDJ)-segments of the immunoglobulin heavy chain, two DLBCL relapse models have been observed: A late (linear) model and early (divergent) model [15]. Data on mutational evolution in DLBCL remains largely unknown due to the lack of tumor biopsies [18]. In the current study, mutation profiles in relapsed DLBCL were analyzed by pair-wise comparison of primary tumor and relapse samples. Despite the limited number of patients with informative WES data, several compelling observations were made.

First, a median of 12.5% of mutations detected in relapse samples was not detected in the primary samples. Our findings are in line with a previous study showing >80% concordance of mutations in 6 out 7 paired R/R samples [38]. However, in 2 of 6 cases we observed a gain >20% in mutations in the relapse sample. These might be truly relapse specific, or failed detections in the primary sample due to heterogeneity. Based on phylogenetic trees by somatic hypermutation and mutational analysis, an increased number of mutations was proposed to be the result of an early divergent relapse clone [15]. The change in mutational load of relapses might be bigger in the presence of spatial heterogeneity, as exemplified by the divergence in acquired mutations between multiple relapse samples in the two cases from the current study. Nevertheless, the observed temporal heterogeneity in DLBCL is relatively low compared to other lymphoproliferative diseases (LPD). In chronic lymphocytic leukemia (CLL) [39,40], follicular lymphoma (FL) [41,42], and mantle cell lymphoma (MCL) [43], relatively large temporal and spatial heterogeneity was observed. In contrast, mutational analysis of circulating cell free tumor DNA in classical Hodgkin lymphoma (HL) indicates a relatively constant mutation profile [44]. 

Secondly, mutations in several known targets of somatic hypermutation were detected as private mutations in relapse samples [35]. Although the most frequent base pair substitution (C:G > T:A) can arise as a consequence of canonical AID activity, it is also the most frequently observed base pair change caused by cyclophosphamide [21,36]. The type and impact of clonal evolution varies depending on the LPD, type of therapy, and involved genes and pathways. For example, CLL patients who relapse after ibrutinib acquire mutations in Bruton Tyrosine Kinase (*BTK*) or phospholipase C-γ2 (*PL*Cγ2) [45], whereas ibrutinib refractory MCL patients acquire mutations within several pathways, including genes of the NF-kB pathway, the mTOR pathway, and epigenetic modifiers [46].

Third, a minority of mutations detected in the primary samples could not be detected in the relapse samples. The low MAF of these mutations is suggestive of sub clonal passenger mutations, a phenomenon that has also been observed in CLL [39]. This data is in line with the study by Morin et al., in which loss of mutations was observed infrequently in cases with VAF < 0.2 [18]. Retention of the vast majority of mutations identified in the primary tumor samples is important for reliable assessment of minimal residual disease (MRD) in free circulating tumor DNA (ctDNA) using targeted approaches [38,47,48]. Focusing on sub clonal passenger mutations might lead to false negative MRD results. Using broad panel based strategies reduces this potential risk and has revealed a success rate of 80–85%. Another main advantage of a broad-panel based ctDNA analysis is that it also allows detection of mutations not observed in the primary biopsy, thus at least partially overcoming the spatial heterogeneity of the tumor sample [38]. Interestingly, ctDNA based analysis of *BTK* revealed emerging mutations in two of three DLBCL patients receiving ibrutinib [38].

Fourth, through pathway analysis we observed enrichment for mutations in genes related to antigen presentation (HLA class I and II molecules (HLA locus), *B2M*, *CALR*) in relapse samples. Although the HLA locus is a highly polymorphic region and somatic mutation calling is prone to errors, the high frequency of mutations in antigen-presentation related genes observed is consistent with previous studies [49]. Approximately 75% of DLBCL exhibit a genetic basis for immune escape [21]. Recently, this type of immune editing has been linked with mutations in *MYD88* and *CD79B* [20]. In DLBCL, mutations in genes involved in immune escape (e.g., B2M and CD58) were associated with increased risk of relapse by selective pressure analysis [12]. Analysis of paired DLBCL samples showed somatic mutations, Indels, or chromosomal deletions targeting CD58 and B2M in five out of seven cases [18]. Mutations in these genes have been postulated as relapse-associated events [15]. Loss of HLA-molecules is frequently observed in DLBCL, and this might have implications for immunotherapy [50]. 

Fifth, we observed mutations in *SOCS1 (5/6)*, *PIM1 (5/6)*, and *MYC (3/6)* in multiple patients, with part of the mutations being relapse specific or enriched. Compared to the reported mutation frequencies in these genes in newly diagnosed DLBCL [51], the frequency seems to be enriched in R/R DLBCL in this study. Based on a mathematical approach, *SOCS1* and *PIM1* mutations were amongst the genes with the highest selective pressure estimates [12]. *PIM1* mutations have been reported in 38% of R/R DLBCL ABC-type cases, and *MYC* mutations in 11% of R/R DLBCL cases [18]. In addition, an enrichment for *SOCS1* and *PIM1* mutations in matched tumor samples was not only observed in R/R DLBCL (4 out of 7 patients), but also in transformed FL (4 out of 7 patients), as well as in relapsed FL (2 out of 7 patients), further supporting the role of mutations in these genes in relation to relapse [38]. In the genomic model of Reddy et al., *PIM1* and *MYC* mutations, but not *SOCS1*, were significantly correlated with decreased survival [19]. This is in line with our observation, which shows a relatively constant MAF for *SOCS1* and an increased MAF for *PIM1* mutations in two of five patients. The dynamics of *PIM1* mutations should be taken into account when treating patients with BTK inhibition, since *PIM1*-stabilizing mutations affect upstream regulators and downstream targets of the NF-kB pathway, decreasing sensitivity of ABC-type DLBCL to BTK inhibition [52]. 

Finally, through pathway analysis we observed enrichment for possible therapy related genes related to trans membrane receptor tyrosine kinases (RTK) and genes involved in the JAK/STAT pathway. Gains of mutations in RTKs was previously observed in matched R/R DLBCL samples [15]. Both SOCS1 and PIM1 converge at the JAK/STAT signaling pathway. While SOCS1 inhibits JAK/STAT signaling, PIM1 expression is correlated with activation of STAT [51,53]. In addition to our observations, loss of the Interleukin 9 receptor (IL9R) locus was previously observed in three out of seven relapsed DLBCL cases [15], and *STAT6* mutations were reported in 36% of R/R GCB-type DLBCL [18], both further implicating a role for the JAK-STAT pathway in relapse.

This study has several limitations. It clearly shows the challenge of obtaining relapse biopsies and good quality genomic DNA from FFPE tissues for WES. Of the 61 initially identified patients, we obtained reliable WES data of representative sequential tumor biopsies for only 6 patients (10%). In half of the relapse cases in our series a biopsy was omitted, and when available, again in half of the cases the biopsy was not sufficient for this type of analysis. Although histological confirmation of R/R DLBCL is advocated, it is often omitted for various reasons. In primary R-CHOP refractory patients hardly any re-biopsies are performed. This is reflected in the current study, in which only half of the patients had histological confirmation. In an attempt to maximize the number of patients in this study, WES was performed on all but one excision biopsy, despite suboptimal DNA quantity and quality. Unfortunately, either primary or R/R samples of 8 out of 14 eligible cases failed library preparation, resulting in sufficient quality data for only 6 patients. Degradation of DNA during fixation and storage might lead to sequencing artifacts, including false positive C:G > T:A substitutions [54]. More than 90% of mutations detected by our pipeline in the primary samples could also be detected in the relapse sample, indicating that our filtering criteria successfully eliminated sequencing artifacts. To avoid such artifacts, it would be better to use fresh frozen samples. However, FFPE is the main mode of storing tissue samples [15,18]. We cannot completely rule out the presence of mutations in genes with low coverage in WES, as exemplified by *FOXO1* [18].

An alternative approach to avoid fixation artifacts applied in more recent studies is the analysis of ctDNA. Initial studies showed the potential of using ctDNA to evaluate clonal evolution in LPDs [42,46,47]. Larger studies using ctDNA analysis could provide a comprehensive view on the mutational evolution of R/R DLBCL. Nonetheless, current ctDNA analysis encompasses preselected lists of target genes, while WES offers the best chance of discovering novel resistance-promoting mutations, especially when moving to more targeted therapy. Implementation of ctDNA analysis in clinical practice requires further standardization for purification and detection of mutations to achieve high sensitivity, especially for detection of MRD. Finally, our study is not powered to address the impact of individual mutations. Nevertheless, recurrent mutations with variable MAF were observed in three genes that all have been implicated with therapy resistance [18,19].

## 5. Conclusions

We show modest temporal heterogeneity between paired tumor samples with the acquisition of new mutations and enrichment of possible therapy resistant related genes. These mutational dynamics should be taken into account when setting up and analyzing biomarker-driven treatment strategies.

## Figures and Tables

**Table 1:** Patient and clinicopathological characteristics of the six patients evaluable for mutational analysis.

Patient Characteristics					Clinicopathological Characteristics							Outcome		
ID	M/F	Age	Stage	IPI	Morphology	COO	Aberrant IHC	FISH	Primary Biopsy	Relapse Biopsy 1	Relapse Biopsy 2	End-of-Treatment	PFS (Months)	OS (Months)
1	F	53	4	3	DLBCL	GCB	n.a.	Inconclusive	Jejunum	Lymph node	-	CR	7	101
2	M	45	2	2	DLBCL	ABC	n.a.	MYC–	Lymph node	Lymph node	-	CR	17	56 ^†^
3	M	65	3	2	DLBCL	GCB	n.a.	MYC–	Soft tissue	Soft tissue	-	PR	7	14 ^†^
4	F	57	3	1	DLBCL	ABC	CD20–	MYC–	Lymph node	Lymph node	-	PD	5	8 ^†^
5	F	57	4	4	HGBCL MYC+/BCL6+ ^#^	Unclassified	CD5+	MYC+ BCL6+	Lymph node A	Lymph node B *	Lymph node C *	n.a.	14	36 ^†^
6	M	79	1	2	DLBCL	GCB	n.a.	MYC–	Soft palate	Skin site A **	Skin site B **	CR	55	55 ^†^

Abbreviations: ABC, activated B-cell; COO, cell-of-origin as determined by the nCounter Lymph2Cx assay; CR, complete remission; DLBCL, diffuse large B-cell lymphoma; FISH, fluorescence in situ hybridization; GCB, germinal center B-cell; IHC, immunohistochemistry; IPI, international prognostic index; n.a., not applicable; OS, overall survival; PFS, progression free survival; PR, partial remission. ^#^ According to the WHO 2017 classification the case is classified as a High grade B-cell lymphoma with MYC and BCL6 rearrangement; * biopsies taken at the different time points; ** biopsies taken at the same time points; ^†^ patient deceased.

## Supplementary Materials

The following are available online at http://www.mdpi.com/2072-6694/10/11/459/s1, Figure S1: Schematic representation of patient and sample selection. Figure S2: Percentage of the target region with indicated read depth (X) across the analyzed samples. Figure S3: Estimated tumor cell percentages. Figure S4: Schematic representation of location and type of mutations in *SOCS1*, *PIM1*, and *MYC*. Figure S5: Loss and increase in mutational load as a variable over time did not show a significant correlation (*ρ*, 0.32; p, 0.71). Figure S6: Distribution of mutations detected only in the relapse samples across the genome showed a random pattern. Figure S7: Global view of mutant allele frequency (MAF) in primary and relapse samples of matched cases. Figure S8: Prevalence of type of base substitutions across the relapse samples. Table S1: Mean read depth (X) of genes known to be mutated in DLBCL at various frequencies in the Cosmic database version 86 across the 14 tumor samples. Table S2: List of 28 genes mutated in 3 or more patients in either the primary or relapse tumor sample, or both. The position and type of mutation and Indels are listed per patient. Table S3: Number of mutations found in either the primary or relapse tumor sample, or both. Table S4: List of kinases (*n* = 18), JAK-STAT signaling (*n* = 7), and glycoprotein (*n* = 73) genes with mutations possibly related to therapy resistance.
