# Supplementary material for: Mutational Evolution in Relapsed Diffuse Large B-Cell Lymphoma
Source: Cancers (Basel). 2018 Nov 20;10(11):459. doi: 10.3390/cancers10110459 (PMC6265691; doi:10.3390/cancers10110459)
Supplement: Supplementary file 1 [file cancers-10-00459-s001.pdf]

Supplementary Materials: Mutational Evolution in Relapsed Diffuse Large B-Cell Lymphoma

Marcel Nijland, Annika Seitz, Martijn Terpstra, Gustaaf W. van Imhoff, Philip M Kluin, Tom van Meerten, Çiğdem Atayar, Léon C. van Kempen, Arjan Diepstra, Klaas Kok and Anke van den Berg

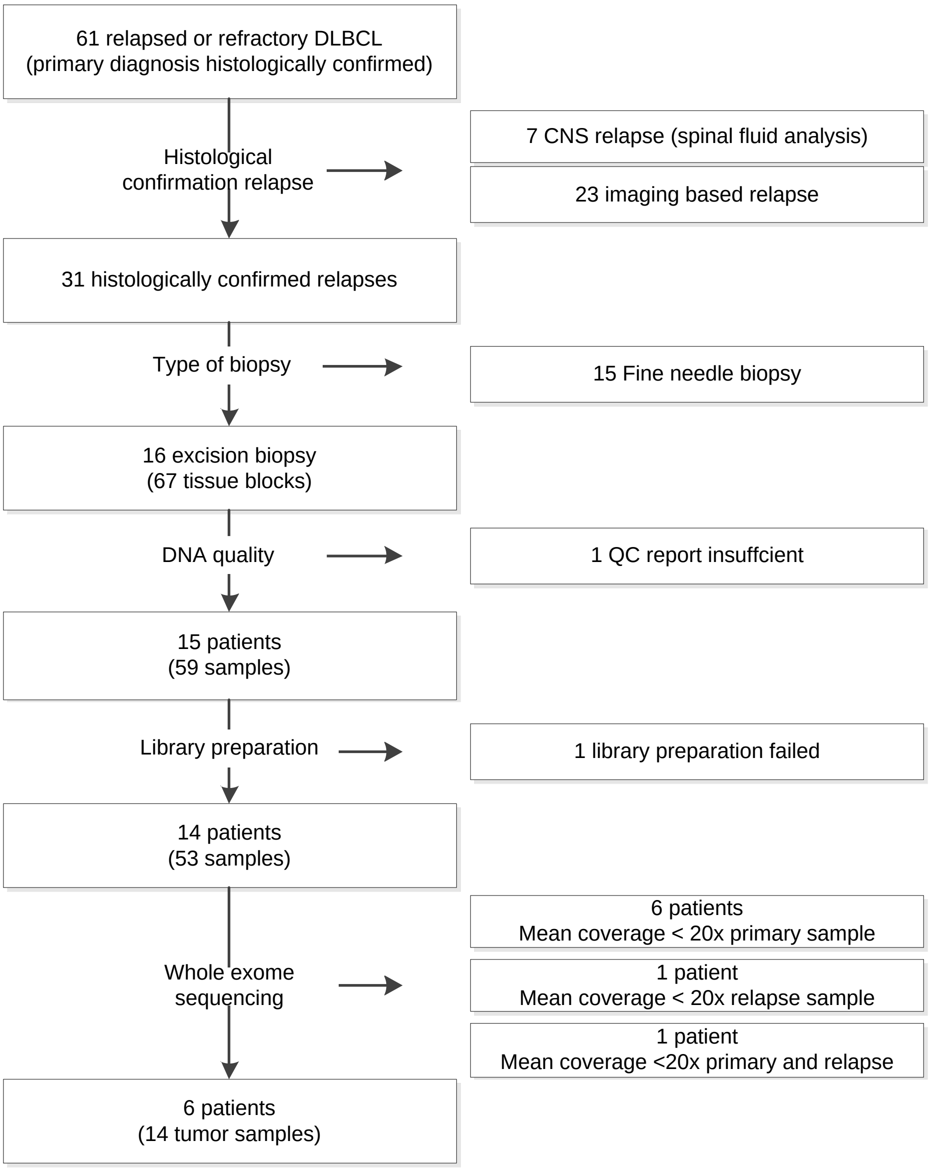

Figure S1. Schematic representation of patient and sample selection. Of the 61 patients, 6 patients had high quality formalin fixed paraffin embedded (FFPE) paired tumor biopsies sufficient for WES analyses. The remainder of the patients were not eligible due to a lack of sufficient tumor tissue or to low coverage.

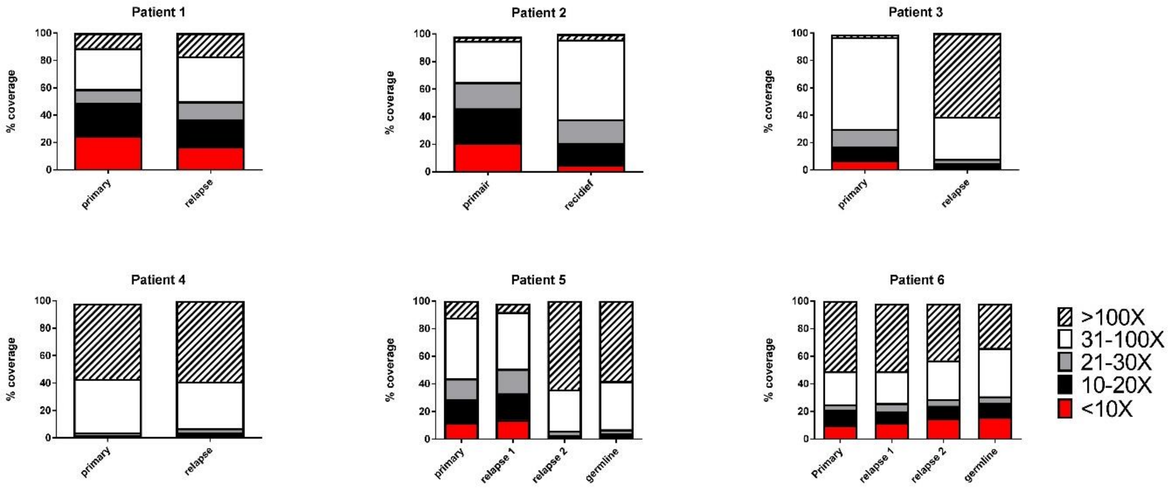

Figure S2. Percentage of the target region with indicated read depth (X) across the analyzed samples.

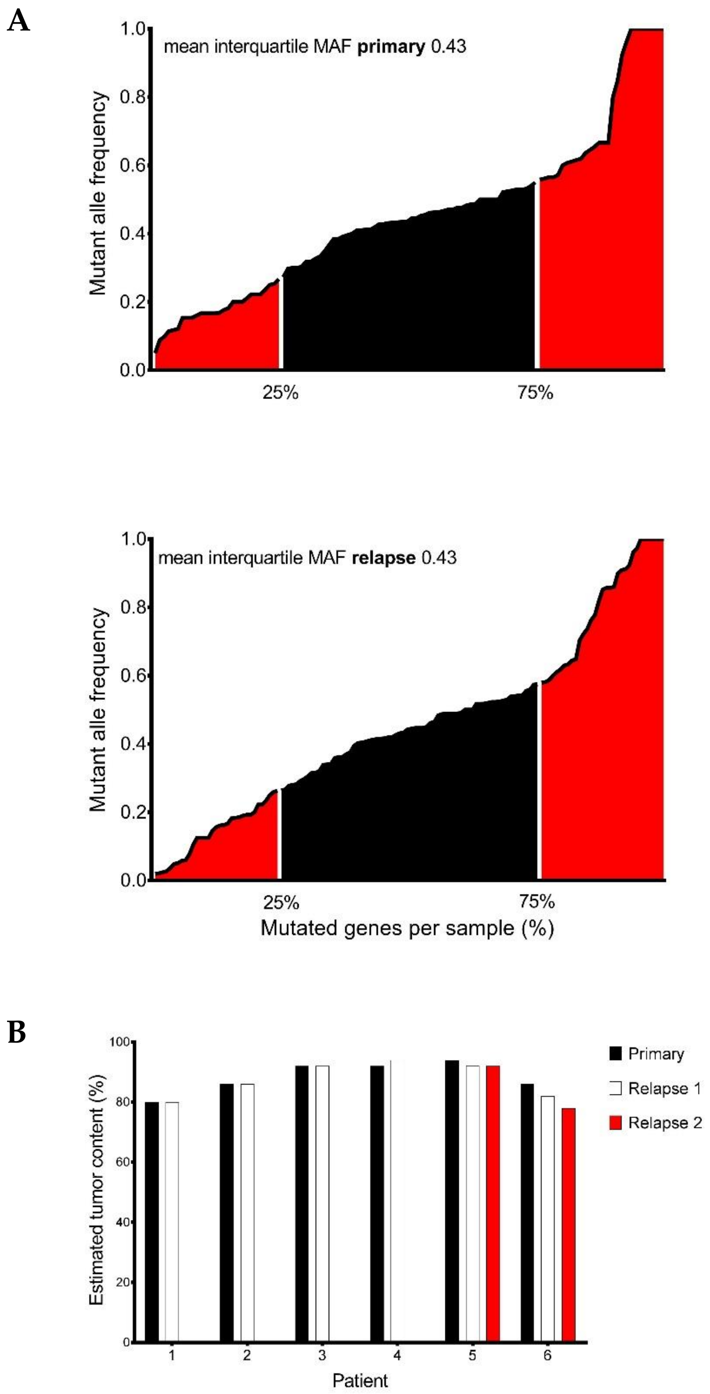

Figure S3. Estimated tumor cell percentages. (**A**) Example of 25–75% interquartile range MAF in two paired biopsies. (**B**) Estimation of the tumor percentages across all samples. The median tumor cell percentage was 90%. There were no significant differences between paired samples.

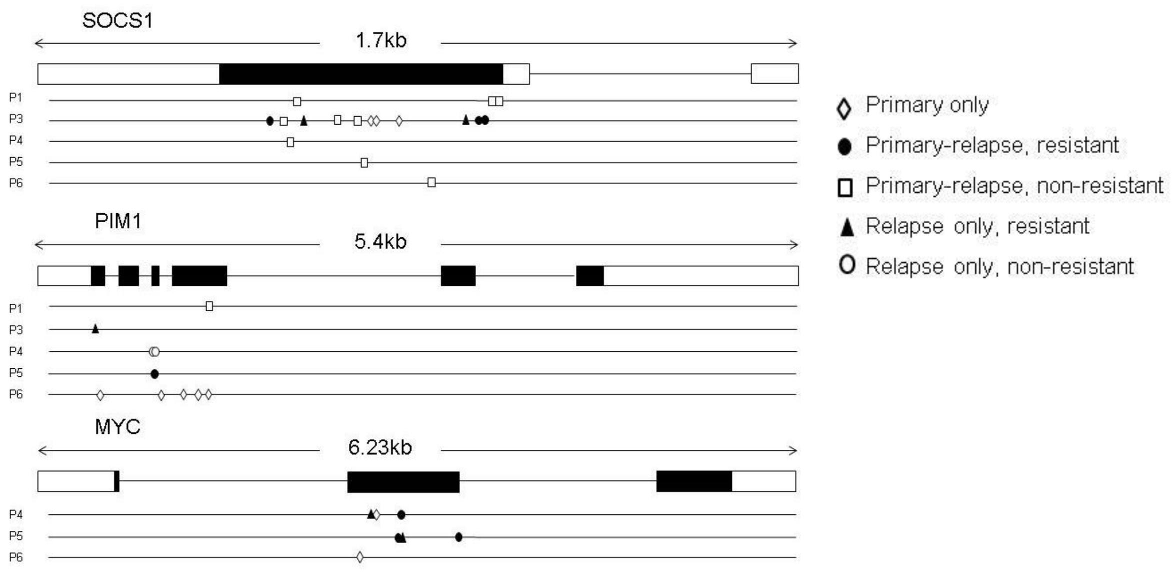

Figure S4. Schematic representation of location and type of mutations in *SOCS1, PIM1*, and *MYC*. In line with the literature, there are no specific hotspots. Symbols represent whether a mutation was found in the primary sample, relapse sample, or both. Mutations with black symbols are possibly related to therapy resistance (mutant alle frequency ≥20% and at least two times higher in the relapse sample compared to the primary tumor).

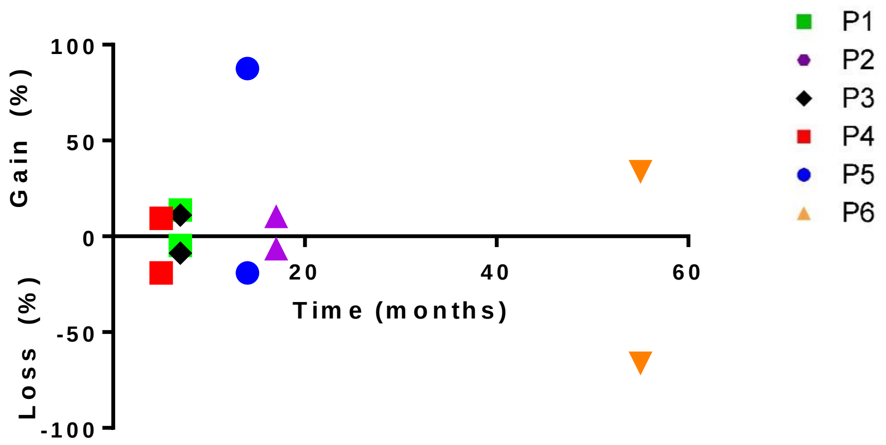

Figure S5. Loss and increase in mutational load as a variable over time did not show a significant correlation (ρ, 0.32; p, 0.71).

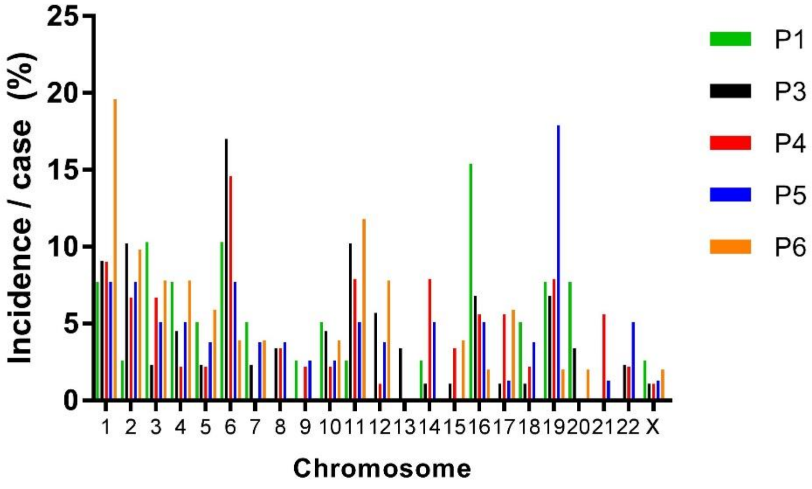

Figure S6. Distribution of mutations detected only in the relapse samples across the genome showed a random pattern. Note: Due to a limited number of mutations, we do not show data for patient 2.

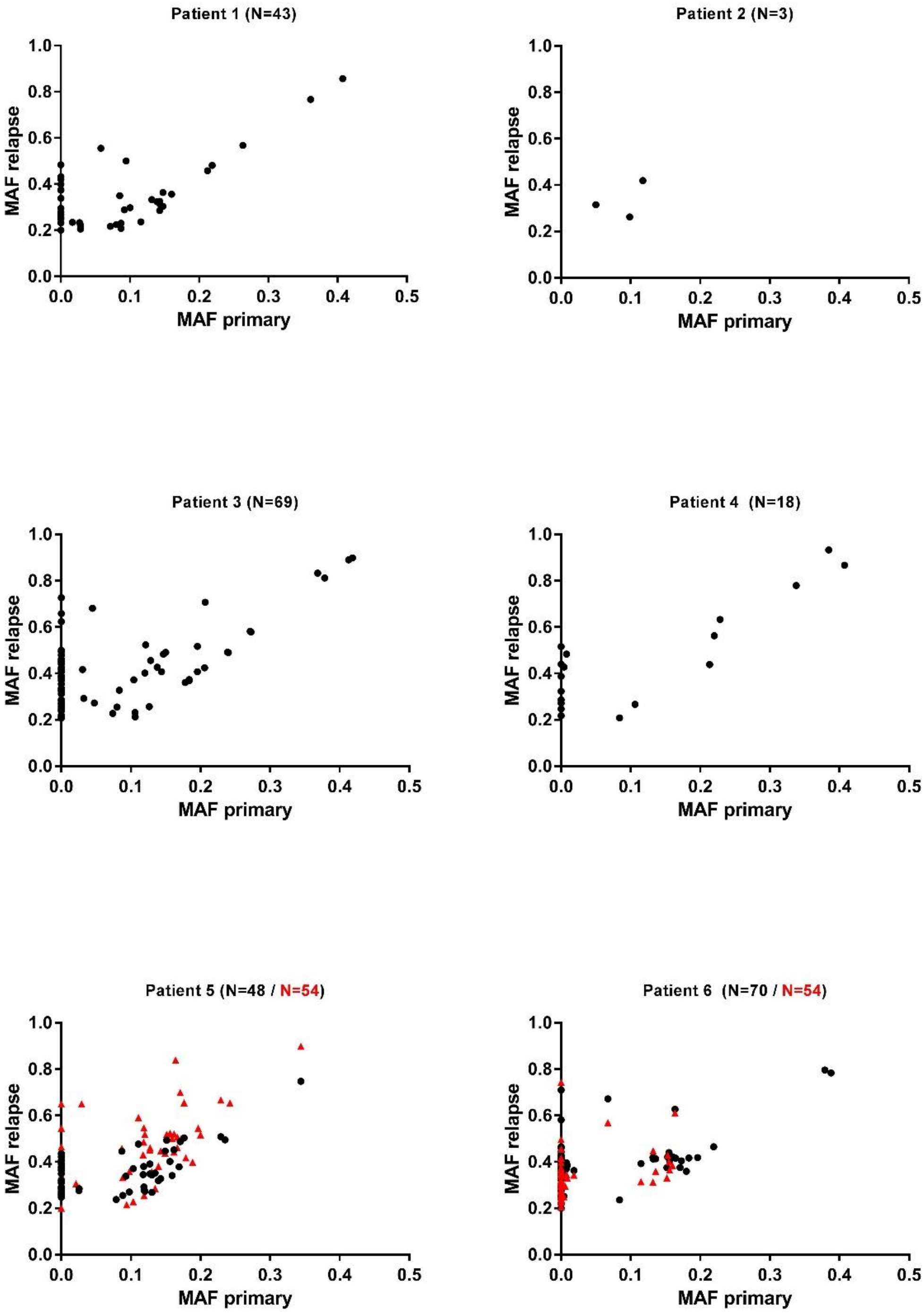

Figure S7. Global view of mutant allele frequency (MAF) in primary and relapse samples of matched cases. Mutations possibly related to therapy resistance have at least 2-fold increased MAF in relapse samples. The red and black dots in the plots of patient 5 and 6 indicate mutations in the 2 different relapse samples. In total there were 374 mutations across 264 genes that were possibly related to therapy resistance.

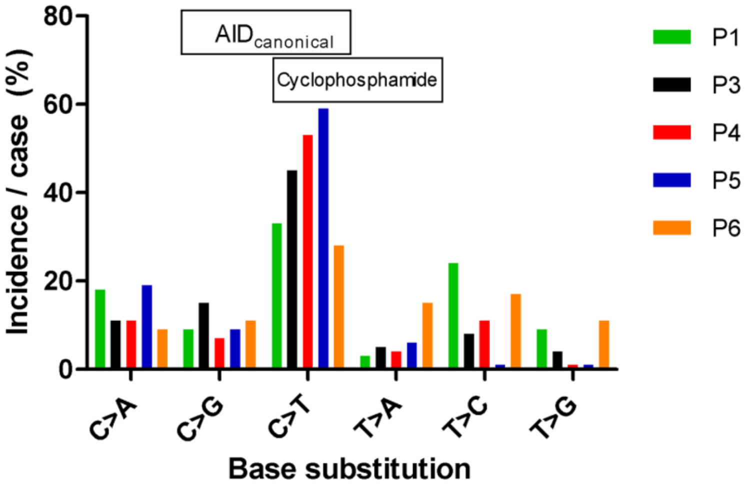

Figure S8. Prevalence of type of base substitutions across the relapse samples. The C:G > T:A base substitution was most frequently observed. This substitution can be caused as a consequence of cyclophosphamide, and to a lesser extent be induced by canonical Activation-Induced Deaminase (AID) activity. Note: Due to a limited number of mutations, no data is shown for patient 2.

Table S1. Mean read depth (X) of genes known to be mutated in DLBCL at various frequencies in the Cosmic database version 86 across the 14 tumor samples.

Gene	Frequency Cosmic (%)	Sample													
		1P	1R	2P	2R	3P	3R	4P	4R	5P	5R1	5R2	6P	6R1	6R2
KMT2D	28	404	399	176	193	201	580	457	1010	277	181	725	783	977	670
EZH2	12	243	189	65	108	163	335	245	267	100	116	506	629 Exon 1 low	633 Exon 1 low	422, Exon 1 low
TNFAIP3	9	246	322	106	117, Exon 8+9 nothing	85, Exon 8+9 nothing	194, Exon 8+9 nothing	456	896	144	103	419	635	409	341
CARD11	8	301, Exon 1+2 nothing	430 Exon 1+2 low	92	213	205	403	212	461	157, first 3 Exons low	72, first 3 Exons low	441	632 Exon 1 low	829 Exon 1 low	607 Exon 1 low
TET2	8	179	216	32	111	99	255	319	474	170	89	427	380 Exon 1+2 low	519 Exon 1+2 low	304 Exon 1+2 low
FOXO1	5,6	Exon 3 no reads	Exon 3 no reads	Exon 3 no reads	Exon 3 no reads	Exon 3 no reads	Exon 3 no reads	Exon 3 no reads	Exon 3 no reads	Exon 3 no reads	Exon 3 no reads	Exon 3 no reads	Exon 3 no reads	Exon 3 no reads	Exon 3 no reads
ZNF521	4,9	155	226	67	92	175	254	246	412	159	84	352	514	502	297
RELN	4,6	92	92	59	108	79	180	118	145	70	61	264	262	284	196
TLN2	4,3	309	352	103	110	123	263	249	545	159	92	300	522	691	369
DMXL1	3,4	142	124	61	78	132	184	177	217	113	82	382	291	267	205
DSP	2,8	209	332	83	137	135	400	325	762	212	98	490	556	711	413
FRMPD1	2,8	137	212	44	110	102	289	299	756	166	92	416	559	651	454
CD79A	2,2	294	300	71	127	111	378	343	556	139	124	369	531	741	361
CSMD3	2,2	113	87	84	96	82	151	133	214	113	71	301	220	216	238
ATM	2,0	197	126	72	119	89	229	221	190	127	72	411	363	327	300
BRAF	2,0	129	144	56	122	261	164	187	204	89	56	307	232	200	205
PTEN	1,8	104	63	79	101	81 Exon 5/9 low	115 Exon 5/9 low	204	147	80 Exon 5 low	80, Exon 5 nothing	80 Exon 5 low	415 Exon 5 low	304 Exon 5 low	320 Exon 5 low
CD79B	1,3	342	389	91	130	95	370	377	894	226	137	561	900	1494	807
FBXO11	1,9	103, Exon 5/13 low	160, Exon 5/13 low	74	108	99	237	269	363	79	74	322	299	248	207
NOTCH1	1,9	353	534	127	144	159	369	309	970	216	133	500	653	841	532
CD83	0,9	199	241	64	120	122	338, Exon 4+5 nothing	546	875	139	130	482	615	627	394

Table S2. List of 28 genes mutated in 3 or more patients in either the primary or relapse tumor sample, or both. The position and type of mutation and Indels are listed per patient.

Patients	Gene	Symbol	P1	P2	P3	P4	P5	P6
5	Pim-1 proto-oncogene, serine/threonine kinase	PIM1	37139198, G/A	-	37138423, G/C	37138793, GACTGGGGAGAGC /G<br>37138790, TC/T	37138804, G/C	37138553, G/C<br>37138956, G/A<br>37139004, G/A<br>37139204, C/G<br>37139063, G/A
5	suppressor of cytokine signaling 1	SOCS1	11349329, CT/C<br>11348873, C/G<br>11349332, C/A	-	11 mutations	11348706, C/G	11349029, C/G	11349162, G/C
4	AHNAK nucleoprotein 2	AHNAK2	105412260, G/C<br>105415607, C/G	-	105412163, C/G<br>105412138, A/G<br>105418391, C/T	105412138, A/G<br>105412163, C/G<br>105412260, G/C<br>105415433, T/C<br>105415431, C/G<br>105415411, G/C<br>105417725, C/G	11 mutations	-
4	major histocompatibility complex, class II, DRB1	HLA-DRB1	32552137, G/A<br>32552132, T/A<br>32552131, C/A<br>32552130, C/A<br>32552091, G/C<br>32552085, G/T<br>32552078, A/T	-	14 mutations	14 mutations	48 mutations	-
3	major histocompatibility complex, class II, DRB5	HLA-DRB5	-	-	32497960, CTT/C<br>32497957, C/CAG<br>32489876, T/A<br>32489881, G/T<br>32487164, G/A<br>32489795, T/A,C<br>32489835, C/G<br>32489731, G/C<br>32497905, G/AT	16 mutations	-	32487165, G/C<br>32489856, C/A<br>32489936, T/A<br>32497962, T/G<br>32497961, T/G<br>32489855, T/A<br>32489877, C/A<br>32489933, T/C<br>32489934, C/G
3	major histocompatibility complex, class I, B	HLA-B	-	-	31324210, G/A<br>31324208, G/T<br>31324057, C/A<br>31324536, T/GA<br>31324601, C/CA	12 mutations	-	31324050, C/T<br>31324576, G/A

3	ankyrin repeat and LEM domain containing 1	ANKLE1	-	-	31324207, AGG/A<br>31324205, G/GCC<br>17397499, GTT/G<br>17397497, GTGTT/G<br>17397481, G/T<br>17397493, G/T<br>17397483, G/T<br>17397353, C/G	17397493, G/T<br>17397483, G/T<br>17397495, GTGTGTT/G<br>17397497, GTGTT/G	17397497, GTGTT/G<br>17397499, GTT/G	-
3	GTPase activating protein	BCR	23523353, G/A	-	23523446, C/T<br>23523445, G/C<br>23523478, G/C<br>23523979, C/A<br>23523635, A/G<br>23523937, A/G<br>23523923, A/C<br>23523266, C/T	-	-	23523586, C/T
3	filaggrin	FLG	-	152278856, T/G<br>152281228, C/G	152281039, G/A<br>152281228, C/G<br>152278706, G/A<br>152284948, G/A	-	-	152276954, G/A<br>152276798, C/T
3	transmembrane phosphatase with tensin homology	TPTE	10906915, C/T	10942924, CCTT/C<br>10943003, C/T<br>10942756, G/A	-	10942756, G/A<br>10942923, G/A	-	-
3	major histocompatibility complex, class II, DQB1	HLA-DQB1	32634302, A/G	-	32632688, T/G<br>32632694, C/AG<br>32632703, G/A	32632688, T/A<br>32632601, G/C	-	-
3	MYC proto-oncogene, bHLH transcription factor	MYC	-	-	-	128750783, G/A<br>128750938, C/T<br>128750713, C/T	128750686, C/T<br>128750683, C/T<br>128751265, G/A	128750680, A/G
3	major histocompatibility complex, class I, C	HLA-C	31238053, G/C	-	31239006, G/T	31239101, G/A,T<br>31237802, A/G<br>31238957, A/C	-	-
3	immunoglobulin lambda like polypeptide 5	IGLL5	-	-	23237597, CCT/C	23230396, TCAGTTGGAAGCA GCCGATCCAGC/T<br>23235946, G/C	-	23230316, T/C<br>23230360, G/A
3	notch 2 N-terminal like A	NOTCH2NL	-	145281656, A/T	-	145281613, C/A<br>145281633, C/A	145273366, C/T<br>145281613, C/A<br>145281633, C/A<br>145281656, A/T<br>145281408, C/T	-

						145273345, T/C<br>145281543, C/T	
3	protein tyrosine phosphatase, receptor type U	PTPRU	-	29602227, G/T	29609217, G/A<br>29587338, G/A	29587407, A/C	-	-
3	Fc fragment of IgG binding protein	FCGBP	40392588, C/T	-	40408268, G/T<br>40368330, C/T	40368321, G/A	-	-
3	collagen type XVIII alpha 1 chain	COL18A1	-	-	-	-	-	-
3	ATPase family, AAA domain containing 3B	ATAD3B	-	1418456, C/CCT<br>1431165, C/T	1430957, G/A<br>1431165, C/T	1425753, T/C	-	-
3	ciliary rootlet coiled-coil, rootletin	CROCC	-	17266536, G/C	17264920, C/T	17264920, C/T	-	-
3	kelch like family member 14	KLHL14	-	-	-	30350086, C/T	30350026, G/A	70314679, A/AAGTAAATTTTTTT<br>30349614, G/A
3	BAI1 associated protein 2 like 2	BAIAP2L2	38483155, T/TTCATGGGTG	38482352, GTGCGGGAGCGGGACTGGCCATCCCAGTACTCCGAGGGTGCTA/G<br>38483155, T/TTCATGGGTG	-	38482352, GTGCGGGAGCGGGACTGGCCATCCCAGTACTCCGAGGGTGCTA/G	-	-
3	PR/SET domain 16	PRDM16	3328089, G/A	-	-	-	3328089, G/A	3328826, G/A
3	NODAL modulator 1	NOMO1	-	-	14969016, T/A	14969016, T/A	-	14969108, C/T
3	sperm associated antigen 17	SPAG17	118584488, C/G	-	118609491, C/A	118548044, G/T	-	-
3	maestro heat like repeat family member 2A	MROH2A	234741285, G/A	-	234740114, TC/T	234698550, C/T	-	-
3	SET domain containing 1B	SETD1B	-	-	122261163, G/A	122260947, G/A	30991144, C/T	-
3	keratin associated protein 1-3	KRTAP1-3	39190954, G/GCAGCAGCTTGGCTGGCAGCAGCTGGTCTCA	-	39190954, G/GCAGCAGCTTGGCTGGCAGCAGCTGGTCTCA	39190954, G/GCAGCAGCTTGGCTGGCAGCAGCTGGTCTCA	-	-

Table S3. Number of mutations found in either the primary or relapse tumor sample, or both.

Patient	Total	Primary Total	Primary Only	Mutual	Relapse Total	Relapse Only	Relapse Only MAF ≥ 0.2	Increase *	Loss #
	n	*n*	*n*	*n*	*n*	*n*	*N* (%)	%	%
**1**	330	290	14	276	316	40	20 (50)	13.8	4.8
**2**	85	77	5	72	80	8	0 (0)	10.4	6.5
**3**	884	796	69	727	815	88	52 (59)	11.1	8.7
**4**	1038	949	59	890	979	89	9 (10)	9.4	6.2
**5**	167	89	17	72	150	78	32 (41)	87.6	19.1
**6**	202	151	100	51	102	51	46 (90)	33.8	66.2
**Median**								12.5	7.6

* Increase of mutations = Primary total / Total; # Loss of mutations = Primary only / Primary total.

Table S4. List of kinases (*n* = 18), JAK-STAT signaling (*n* = 7), and glycoprotein (*n* = 73) genes with mutations possibly related to therapy resistance.

Gene	Symbol
**Kinases**	
A-kinase anchoring protein 1	AKAP1
BCR, RhoGEF and GTPase activating protein	BCR
NAD kinase	NADK
NUAK family kinase 1	NUAK1
Pim-1 proto-oncogene, serine/threonine kinase	PIM1
checkpoint kinase 1	CHEK1
cyclin dependent kinase inhibitor 2A	CDKN2A
diacylglycerol kinase eta	DGKH
diacylglycerol kinase zeta	DGKZ
doublecortin like kinase 1	DCLK1
dual specificity tyrosine phosphorylation regulated kinase 1B	DYRK1B
nucleolar protein 9	NOL9
phosphatidylinositol-4,5-bisphosphate 3-kinase catalytic subunit alpha	PIK3CA
phosphorylase kinase catalytic subunit gamma 1	PHKG1
receptor tyrosine kinase like orphan receptor 1	ROR1
serum/glucocorticoid regulated kinase 1	SGK1
tau tubulin kinase 2	TTBK2
tyrosine kinase 2	TYK2
**JAK-STAT signaling pathway**	
Pim-1 proto-oncogene, serine/threonine kinase	PIM1
interleukin 13 receptor subunit alpha 1	IL13RA1
phosphatidylinositol-4,5-bisphosphate 3-kinase catalytic subunit alpha	PIK3CA
suppressor of cytokine signaling 1	SOCS1
suppressor of cytokine signaling 2	SOCS2
tyrosine kinase 2	TYK2
v-myc avian myelocytomatosis viral oncogene homolog	MYC
**Glycoprotein**	
ATP binding cassette subfamily A member 12	ABCA12
ATPase Na+/K+ transporting subunit beta 2	ATP1B2
BMP/retinoic acid inducible neural specific 3	BRINP3
FAT atypical cadherin 3	FAT3
GDNF family receptor alpha like	GFRAL
HGF activator	HGFAC
MAM domain containing glycosylphosphatidylinositol anchor 2	MDGA2
NODAL modulator 1	NOMO1
NUS1 dehydrodolichyl diphosphate synthase subunit	NUS1
UDP glucuronosyltransferase family 2 member B7	UGT2B7
activating transcription factor 6 beta	ATF6B
adenylate cyclase 6	ADCY6
astrotactin 1	ASTN1
calreticulin	CALR
carboxypeptidase Z	CPZ
cholinergic receptor muscarinic 3	CHRM3
chromosome 17 open reading frame 77	C17orf77
collagen type VII alpha 1 chain	COL7A1
diacylglycerol lipase alpha	DAGLA
dynein axonemal heavy chain 10	DNAH10
endothelial cell adhesion molecule	ESAM
fibrinogen beta chain	FGB
follicle stimulating hormone receptor	FSHR

galectin 3 binding protein	LGALS3BP
gamma-aminobutyric acid type A receptor pi subunit	GABRP
glutamate ionotropic receptor kainate type subunit 2	GRIK2
glycine receptor beta	GLRB
glycoprotein 2	GP2
glycosyltransferase 8 domain containing 1	GLT8D1
histamine receptor H4	HRH4
histone cluster 1 H2B family member m	HIST1H2BM
histone cluster 2 H2B family member f	HIST2H2BF
hyaluronan binding protein 2	HABP2
immunoglobulin superfamily member 3	IGSF3
interleukin 13 receptor subunit alpha 1	IL13RA1
interleukin 4 induced 1	IL4I1
keratin 18	KRT18
killer cell immunoglobulin like receptor, three Ig domains X1	KIR3DX1
laminin subunit gamma 2	LAMC2
lysozyme like 2	LYZL2
lysyl oxidase like 3	LOXL3
macrophage expressed	MPEG1
melanoma cell adhesion molecule	MCAM
netrin 5	NTN5
neuritin 1 like	NRN1L
olfactory receptor family 14 subfamily A member 16	OR14A16
olfactory receptor family 2 subfamily T member 34	OR2T34
olfactory receptor family 6 subfamily T member 1	OR6T1
olfactory receptor family 8 subfamily I member 2	OR8I2
olfactory receptor family 8 subfamily K member 1	OR8K1
podocalyxin like	PODXL
potassium sodium-activated channel subfamily T member 2	KCNT2
procollagen-lysine,2-oxoglutarate 5-dioxygenase 2	PLOD2
proprotein convertase subtilisin/kexin type 1	PCSK1
proteasome subunit beta 1	PSMB1
protocadherin alpha 5	PCDHA5
receptor tyrosine kinase like orphan receptor 1	ROR1
roundabout guidance receptor 1	ROBO1
semaphorin 4A	SEMA4A
signal regulatory protein beta 1	SIRPB1
sodium voltage-gated channel alpha subunit 3	SCN3A
solute carrier family 22 member 6	SLC22A6
solute carrier family 4 member 4	SLC4A4
somatostatin receptor 5	SSTR5
stabilin 2	STAB2
synaptotagmin 1	SYT1
taste 1 receptor member 3	TAS1R3
taste 2 receptor member 50	TAS2R50
tetraspanin 17	TSPAN17
transforming growth factor beta receptor 3	TGFBR3
transmembrane protein 123	TMEM123
tryptase alpha/beta 1	TPSAB1
v-myc avian myelocytomatosis viral oncogene homolog	MYC
